# 3D Cultures of Parkinson's Disease‐Specific Dopaminergic Neurons for High Content Phenotyping and Drug Testing

**DOI:** 10.1002/advs.201800927

**Published:** 2018-11-20

**Authors:** Silvia Bolognin, Marie Fossépré, Xiaobing Qing, Javier Jarazo, Janez Ščančar, Edinson Lucumi Moreno, Sarah L. Nickels, Kobi Wasner, Nassima Ouzren, Jonas Walter, Anne Grünewald, Enrico Glaab, Luis Salamanca, Ronan M. T. Fleming, Paul M. A. Antony, Jens C. Schwamborn

**Affiliations:** ^1^ Luxembourg Centre for Systems Biomedicine University of Luxembourg 6 avenue du Swing Belvaux L‐4367 Luxembourg; ^2^ Braingineering Technologies SARL 9 avenue des Hauts‐Forneaux Esch‐sur‐Alzette L‐4362 Luxembourg; ^3^ Department of Environmental Sciences Jožef Stefan Institute Jamova 39 1000 Ljubljana Slovenia; ^4^ Institute of Neurogenetics University of Lübeck 23562 Lübeck Germany

**Keywords:** high‐content imaging, microfluidics, Parkinson's disease, stem cells

## Abstract

Parkinson's disease (PD)‐specific neurons, grown in standard 2D cultures, typically only display weak endophenotypes. The cultivation of PD patient‐specific neurons, derived from induced pluripotent stem cells carrying the LRRK2‐G2019S mutation, is optimized in 3D microfluidics. The automated image analysis algorithms are implemented to enable pharmacophenomics in disease‐relevant conditions. In contrast to 2D cultures, this 3D approach reveals robust endophenotypes. High‐content imaging data show decreased dopaminergic differentiation and branching complexity, altered mitochondrial morphology, and increased cell death in LRRK2‐G2019S neurons compared to isogenic lines without using stressor agents. Treatment with the LRRK2 inhibitor 2 (Inh2) rescues LRRK2‐G2019S‐dependent dopaminergic phenotypes. Strikingly, a holistic analysis of all studied features shows that the genetic background of the PD patients, and not the LRRK2‐G2019S mutation, constitutes the strongest contribution to the phenotypes. These data support the use of advanced in vitro models for future patient stratification and personalized drug development.

## 1 Introduction

The identification of promising drug candidates in preclinical research, as well as personalized precision medicine, is hampered by the lack of sufficiently representative in vitro models. This is particularly true in the case of Parkinson's disease (PD), a complex neurodegenerative disorder where the most studied cells, associated to the onset of motor dysfunctions, are the dopaminergic neurons of the *substantia nigra* in the midbrain.^1^ PD is a disorder for which animal models are not sufficiently predictive of the human response. The difficulty of capturing the multifactorial nature of the disease in conventional in vitro models and the excessive reliance on animal models partly explain the disappointingly high failure rate of new candidate molecules in clinical trials.^1^, ^2^ New technological advancements have failed to translate into successful curative pharmacological options and no definitive disease‐modifying therapy is currently available.

In this scenario, human induced pluripotent stem cells (iPSCs) represent a promising tool for the generation of relevant human in vitro models, due to their ability to differentiate into any cell type of the body.^3^ However, the use of iPSC technology alone is often not sufficient to account for all the limitations of modeling complex diseases in a dish. Standard 2D cell culture systems do not offer an ideal set‐up to study highly ramified cells such as neurons. In 2D cultures, the dendrites and growth cones are unrealistically flattened, limiting the acquisition of full cellular functionality, and the cellular microenvironment is poorly modelled. It has been shown that cells in a 3D in vitro setting are subjected to mechanostructural cues which bring them closer to physiological conditions.^4^ Surrounded by matrix surrogates, cells experience a more physiological equilibration and transport of soluble factors.^4^, ^5^ Notably, several groups have reported significantly different gene and protein expression profiles when comparing 2D and 3D cultures.^6^ Cells cultured in 2D showed ≈30% of differentially expressed genes compared to cells in vivo.^7^ It is not surprising that the cellular responses to drug administration in 3D were closer to in vivo than 2D cultures.^8^ This superior metabolic competent system holds the potential to enable pharmacological studies in personalized human‐derived in vitro models at very early stages of the drug discovery pipeline. In the context of Alzheimer's disease, it was reported that 3D cultivation of gene‐edited stem cell‐derived neurons, expressing mutations responsible for the familial form of the pathology, successfully recapitulated all key known neuronal hallmarks of the disease for the first time in an in vitro system.^9^

We have recently shown that iPSCs‐derived neuroepithelial stem cells can be used for the generation of midbrain‐specific dopaminergic neurons in a 3D microfluidics device.^10^ The most common pathogenic mutation causing autosomal‐dominant PD is the G2019S mutation in the leucine‐rich‐repeat‐kinase‐2 (LRRK2).^11^ The LRRK2‐associated familial form is pathologically and clinically indistinguishable from idiopathic PD.^12^ Remarkably, wild‐type LRRK2 was also found to be relevant to the etiology of sporadic PD, as genome‐wide association study analyses indicated that common variations around LRRK2 can modulate the risk of developing the disease.^13^ Mutated LRRK2 has been reported to alter several cellular pathways including cell proliferation, protein trafficking, and cytoskeletal integrity.^14^ However, due to its two enzymatic and several interaction domains, the contribution of LRRK2 to the pathogenic mechanism causing neuronal degeneration remains elusive.^15^

Here, we demonstrated that 2D cell culture systems, without using stressor molecules, did not present robust endophenotypes affecting dopaminergic neurons. On the contrary, 3D cultures elicited intrinsic time‐dependent dopaminergic degeneration due to the LRRK2‐G2019S mutation. We also showed that LRRK2‐G2019S caused mitochondrial abnormalities and cell death in young neurons. Interestingly, the administration of the LRRK2 inhibitor 2 (Inh2) rescued only some, but not all phenotypes. This indicates that other genetic factors, in addition to the LRRK2‐G2019S mutation, contribute to these phenotypes. A holistic analysis of all the analyzed features showed that the genetic background of the PD patients, and not the LRRK2‐G2019S mutation, constituted the strongest contribution to the phenotypes. Our high‐content image analysis platform also allowed for the identification of key LRRK2‐G2019S‐dependent and independent phenotypes, which can be used to test the effect of potential disease‐modifying compounds in a high‐throughput manner.

## 2 Results

### 2.1 Time‐Dependent Alteration of Dopaminergic Neuron Differentiation Associated to LRRK2‐G2019S

**Figure 1:**
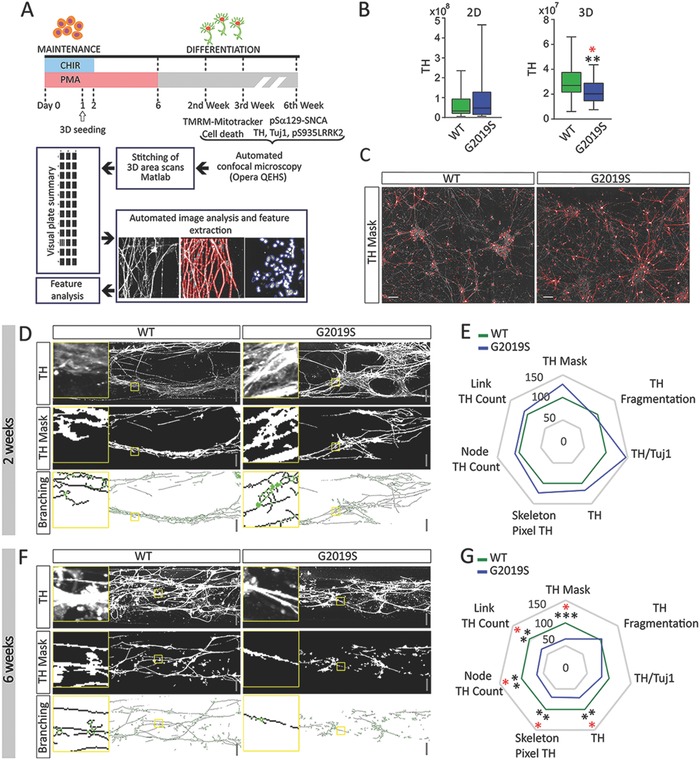
LRRK2‐G2019S dependent phenotype was enhanced in 3D conditions. A) Schematic representation of the experimental pipeline used in the study. Image acquisition, segmentation, feature extraction, and data analysis were all automated. B) Amount of TH^+^ pixels in 2D and 3D cultures after 6w of neuronal differentiation. Values represent means with whiskers from min to max (number of wells in 2D: LRRK2‐WT 48, LRRK2‐G2019S 39; number of bioreactors in 3D: LRRK2‐WT 48, LRRK2‐G2019S 34). C) Representative maximum intensity projection of confocal images of LRRK2‐WT and LRRK2‐G2019S neurons after 6w in 2D showing TH mask after segmentation (red line) superimposed on TH raw channel. D) Representative confocal images of TH^+^ cells and consequent TH skeleton mask and branching rendering after 2w. E) Radar plot showing several features extracted from TH segmentation after 2w (number of bioreactors: LRRK2‐WT 31, LRRK2‐G2019S 32). F) Representative confocal images of TH^+^ cells and consequent TH skeleton mask and branching rendering after 6w. G) Radar plot showing several features extracted from TH segmentation after 6w (number of bioreactors: LRRK2‐WT 48, LRRK2‐G2019S 34). Scale bars 100 µm. In all cases, *p*‐values are calculated using Mann Whitney test and they are adjusted (red) or not (black) according to Benjamini–Hochberg (number of total features assed 18), *p ≤ 0.05, **p ≤ 0.01, ***p ≤ 0.001. For the 3D analysis, 420 fields at 20× magnification were acquired for each bioreactor (21 fields for 20 planes). For the 2D analysis, 45 fields at 20× magnification were acquired for each well (15 fields for 3 planes). Each data point corresponds to a bioreactor.

The present study utilized eight human iPSC lines (Figure S1A, Supporting Information) from which human neuroepithelial stem cell lines (hNESCs) were derived according to published protocols.^16^ We used hNESC lines from two PD patients carrying the LRRK2‐G2019S mutation and from two healthy individuals. In the patient lines, the mutation was corrected to wild‐type sequence, while in the iPSC lines from healthy individuals the wild‐type sequence was replaced by the mutated form (isogenic cell line pairs). Sequencing was performed to confirm correct editing (Figure S1B, Supporting Information). All eight hNESC lines used in the study were checked for expression of neural stem cell markers SOX1, SOX2, and Nestin (Figure S1C, Supporting Information). In agreement with previous reports, the neuronal differentiation protocol generated a mixed population of neural cells, including about 30% of dopaminergic neurons.^10^, ^16^ The experiment pipeline of the study is shown in **Figure** **1**A.

Initially, neuronal differentiation of the hNESC lines was conducted in parallel in 2D and 3D. We used the exact same Matrigel and media cocktails for both conditions, therefore all observed differences are attributed to the different environments (2D vs 3D). Six weeks after the induction of neuronal differentiation the amount of TH^+^ dopaminergic neurons was significantly reduced in LRRK2‐G2019S mutation lines compared to LRRK2‐G2019S, but only in 3D culture (Figure 1B,C). This represents the first evidence for a disease‐specific and 3D‐specific phenotype in our experimental setup.

Next, we decided to focus only on 3D cultures and to better resolve the temporal dynamics of the observed defect. We evaluated the branching complexity of TH^+^ cells after 2 and 6 weeks (w) of differentiation in 3D (Figure 1D,E (2w); Figure 1F,G (6w)). In the 2w cultures, no significant differences between LRRK2‐G2019S and LRRK2‐WT lines were detected (Figure 1E). However, after 6w, we observed a significant decrease in the amount of TH^+^ pixels (TH). Features describing the dopaminergic arborization complexity such as skeleton pixels TH, node, and link count also decreased (Figure 1G, **Table** **1**, and Figure S2 of the Supporting Information summarize the features assessed in the analysis). This highlights a time‐dependent selective degeneration of dopaminergic neurons carrying LRRK2‐G2019S. We also analyzed the levels of α‐synuclein phosphorylation at serine 129 (pS129αSNCA) and of LRRK2 at serine 935 (pS935LRRK2) after either 2w (Figure S1D, Supporting Information) or 6w (Figure S1E, Supporting Information). The pS129αSNCA signal was unchanged at both time points, but pS935LRRK2 levels were increased after 2w in LRRK2‐G2019S lines.

**Table 1:**
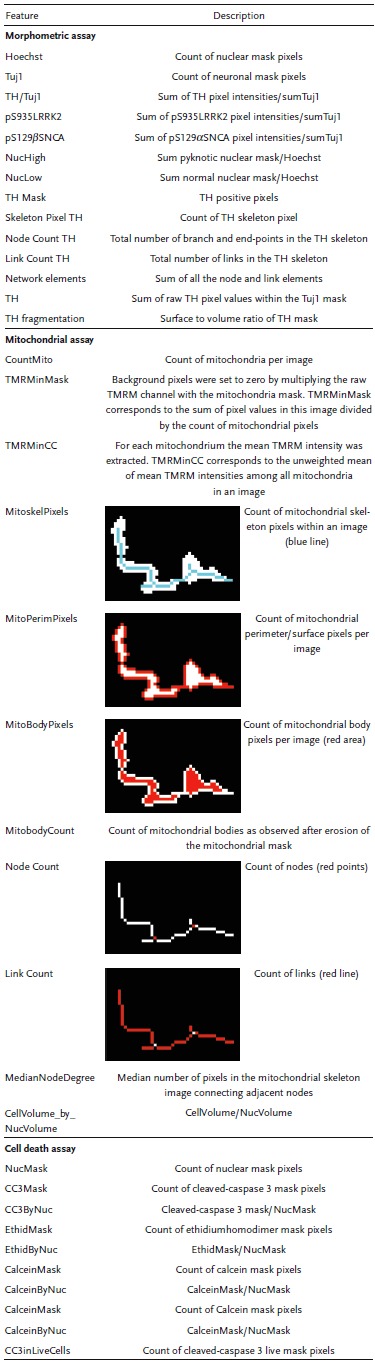
Features from image analysis

### 2.2 LRRK2‐G2019S Induced Mitochondrial Abnormalities and Cell Death in Developing Neurons

**Figure 2:**
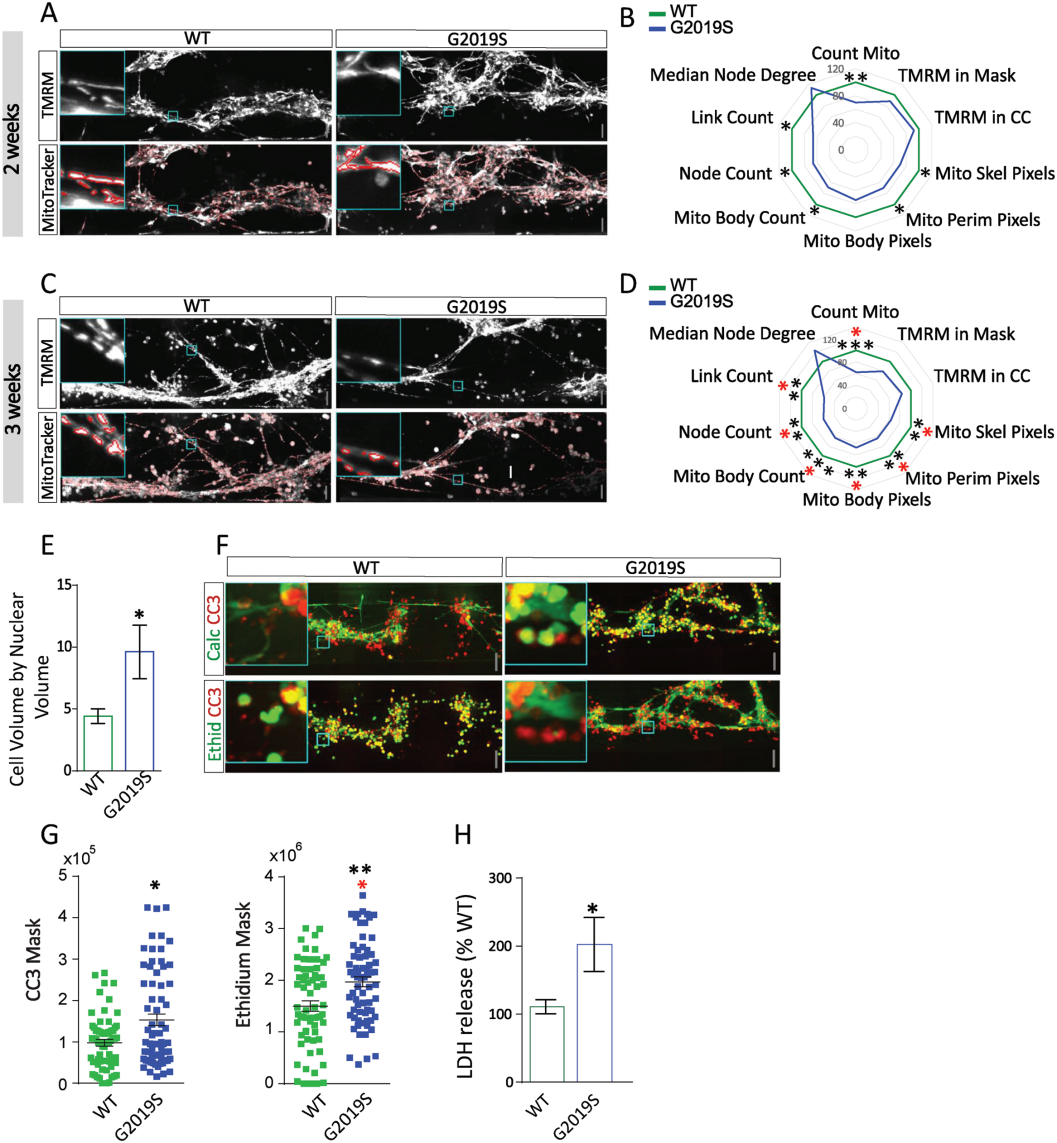
LRRK2‐G2019S induced early mitochondrial dysfunction and cell death. A) Representative maximum intensity projection of confocal images of LRRK2‐WT and LRRK2‐G2019S neurons after 2w showing TMRM raw channel, and segmented mitochondria (red line) superimposed on Mitotracker green channel. B) Radar plot showing several features extracted from mitochondrial segmentation after 2w (number of bioreactors: LRRK2‐WT 68, LRRK2‐G2019S 63). C) Representative maximum intensity projection of confocal images of LRRK2‐WT and LRRK2‐G2019S neurons after 3w showing TMRM raw channel, and segmented mitochondria (red line) superimposed on Mitotracker green channel. D) Radar plot showing several features extracted from the mitochondrial segmentation after 3w (number of bioreactors: LRRK2‐WT 64, LRRK2‐G2019S 61). E) Bar graph showing cell volume by nuclear volume after 3w. F) Representative maximum intensity projection of confocal images of LRRK2‐WT and LRRK2‐G2019S neurons after 2w showing staining for Calcein/cleaved‐caspase 3 (CC3), and ethidium homodimer (EH)/CC3. G) Viability‐related features extracted after 2w of neuronal differentiation: CC3 mask and EH mask (number of bioreactors: LRRK2‐WT 68, LRRK2‐G2019S 66). In all cases, *p*‐values are calculated using Mann Whitney test and they are adjusted (red) or not (black) according to Benjamini–Hochberg, *p ≤ 0.05, **p ≤ 0.01, ***p ≤ 0.001. The number of total features assesed was 21 for (A)–(E) and 9 for (F)–(G). For the analysis in (B) and (D), 63 fields were acquired for each bioreactor (9 fields for 7 planes at 20× magnification). For panel **G**, 420 fields at 20× magnification were acquired for each bioreactor (21 fields for 20 planes at 20× magnification). Each data point corresponds to a bioreactor. H) LDH‐increased release in LRRK2‐G2019S compared to LRRK2‐WT lines after 3w. The experiment was performed nine times, in quadruplicate, in LRRK2‐WT and LRRK2‐G2019S lines. Technical replicates were averaged. Values represent means ±SEM, *p*‐value is calculated using Mann Whitney test *p ≤ 0.05. Scale bars 100 µm.

As 6w old neurons showed LRRK2‐G2019S‐induced alterations in survival and neuronal complexity, we were interested in identifying the potentially underlying upstream mechanisms. Thus, we performed microarray gene expression analysis of hNESC from six healthy individuals and six PD patients carrying the LRRK2‐G2019S mutation (Figure S3A, Supporting Information). Microarray gene expression analysis showed alterations associated with mitochondrial genes, derived from the MitoCarta database of genes encoding proteins with strong support of mitochondrial localization.^17^ A subset of the most significantly altered mitochondrial genes according to the RankProduct method^18^ is shown in the heat map in Figure S3B (Supporting Information). Based on these gene expression data and on several publications highlighting the contribution of mitochondrial alterations to PD,^19^ we tested the cultures for signs of mitochondrial abnormalities at 2w and 3w of neuronal differentiation. In order to understand which mechanisms lead to degeneration, we chose time points before the TH^+^ phenotype became apparent (6w). We performed live imaging with tetramethylrhodamine, methyl ester (TMRM) and MitoTracker Green (**Figure** **2**A,C). The number of mitochondria (MitoCount) was lower at 2w in LRRK2‐G2019S than in LRRK2‐WT neurons (Figure 2B). Mitochondrial morphometric features such as skeleton, perimeter, body pixels, and complexity of the network (number of links and nodes) were also decreased in LRRK2‐G2019S neurons compared to LRRK2‐WT (see Table 1 for the analyzed features). Importantly, in 2D cultures using the same image analysis algorithms, we did not detect any significant alteration (Figure S3C,D, Supporting Information). After 3w of neuronal differentiation, we observed a further progressive reduction in mitochondrial number, perimeter, and complexity (Figure 2D). This implies a detrimental effect of the LRRK2‐G2019S mutation on key components of mitochondrial machinery without impairing mitochondrial membrane potential, consistent with previous studies in fibroblasts.^20^ The ratio between cytoplasmic and nuclear volumes at 3w was significantly increased in LRRK2‐G2019S lines compared to controls (Figure 2E). We hypothesized that this might be an indicator of nucleus shrinking resulting from cell death triggers. Accordingly, we then sought to analyze the activation of cleaved‐caspase 3 (CC3) at similar points. We observed that the time window for increased CC3 activation was at 2w (Figure 2F,G), while the levels at 3w were comparable between LRRK2‐G2019S and LRRK2‐WT (data not shown). The total amount of dead cells as detected by ethidium homodimer (EH) was increased in LRRK2‐G2019S lines compared to LRRK2‐WT. The levels of CC3 (CC3 Mask) were increased in LRRK2‐G2019S compared to LRRK2‐WT lines. Coherently, lactate dehydrogenase (LDH) release into the media was increased in 3w differentiating LRRK2‐G2019S neurons as compared to LRRK2‐WT (Figure 2H). The progressive decrease of extracellular Zn levels in media outflow of LRRK2‐G2019S compared to LRRK2‐WT lines might further support the observed neuronal death (Figure S1F, Supporting Information). This decrease might also be due to a reduction of Zn‐containing vesicles (see review^21^). Taken together, these results indicate that LRRK2‐G2019S neurons are more prone to mitochondrial abnormalities and activation of apoptotic pathways.

### 2.3 Kinase Inhibitor Inh2 Rescued LRRK2‐G2019S Dependent Dopaminergic Phenotype

**Figure 3:**
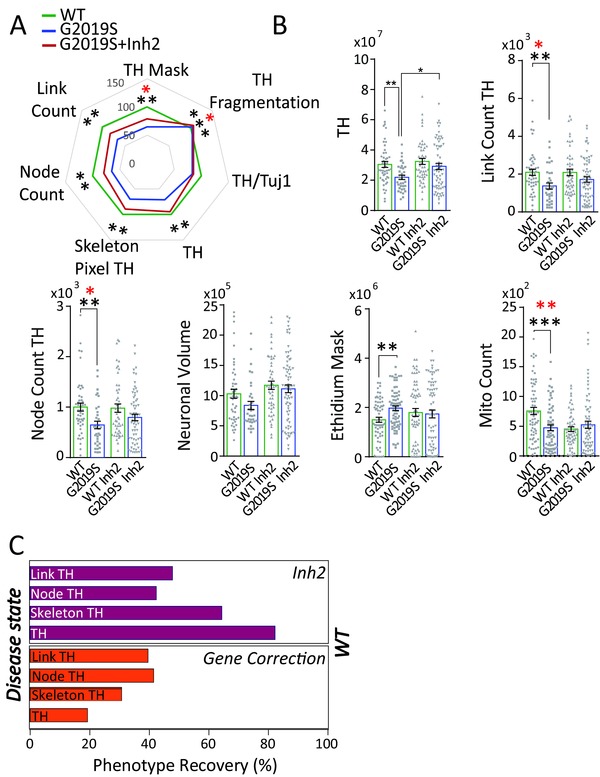
Pharmacological and genetic amelioration of LRRK2‐G2019S‐induced morphometric defects. A) Radar plot showing the rescue of LRRK2‐G2019S‐induced phenotype following Inh2 administration for 6 weeks. Inh2 increased TH levels and ameliorated branching complexity. *p*‐values are calculated using Kruskal–Wallis test. They are adjusted (red) or not (black) according to Benjamini–Hochberg (number of total features assed 18), *p ≤ 0.05, **p ≤ 0.01, ***p ≤ 0.001. The conditions tested are LRRK2‐WT, LRRK2‐G2019S, LRRK2‐G2019S Inh2, and LRRK2‐WT Inh2 (not displayed in the radar plot). B) Bar graphs showing, TH volume, link count TH, node count TH, and neuronal volume after 6w (number of bioreactors: LRRK2‐WT 48, LRRK2‐G2019S 34, LRRK2‐WT Inh2 49, LRRK2‐G2019S Inh2 64). Ethidium mask after 2 weeks (number of bioreactors: LRRK2‐WT 66, LRRK2‐G2019S 68, LRRK2‐WT Inh2 64, LRRK2‐G2019S Inh2 71) and mitochondrial count after 3w (number of bioreactors: LRRK2‐WT 64, LRRK2‐G2019S 61, LRRK2‐WT Inh2 57, LRRK2‐G2019S Inh2 66) are also shown. Bars represent means ± SEM, each dot represents a bioreactor. *p*‐values are calculated using Dunn's post hoc test and they are adjusted (red) or not (black) according to Benjamini–Hochberg (number of conditions compared), *p ≤ 0.05, **p ≤ 0.01, ***p ≤ 0.001. Post hoc analysis was performed on the following pairs: LRRK2‐WT/ LRRK2‐G2019S, LRRK2‐G2019S/LRRK2‐G2019S Inh2. For the analysis, 420 fields at 20× magnification were acquired for each bioreactor (21 fields for 20 planes). C) Bar graphs showing the rescue of selected features achieved with gene editing or Inh2 administration performed by bootstrapping.

Based on the established assays and observed phenotypes, we ran a proof‐of‐concept drug testing screen in our 3D system. We tested the ability of the LRRK2 kinase inhibitor Inh2^22^ to rescue the described phenotypes. After testing concentrations up to 1.5 × 10^−6^ m (data not shown), 0.5 × 10^−6^ m was selected for all experiments in this study. The administration of Inh2 for 6 weeks ameliorated some of the phenotypes observed in the dopaminergic neurons (TH, nodes, links, skeleton TH) (**Figure** **3**A,B). Importantly, the selective degeneration of TH^+^ dopaminergic neurons in LRRK2‐G2019S lines was ameliorated by Inh2 (TH). The restoration was further evidenced by detection of increased link count upon Inh2 administration (Figure 3B). Neuronal volume (Tuj1^+^ cells) increased after treatment in LRRK2‐G2019S lines, indicating a general neuroprotective effect on nondopaminergic neurons. Notably, treatment with Inh2 was not effective in rescuing increased cell death at 2w (Ethidium Mask) or mitochondrial defects (mitochondrial count) at 3w. This suggests Inh2 corrected only some of the pathways affected by LRRK2‐G2019S. Focusing on TH^+^ morphometric analysis, we assessed Inh2 rescue effects by setting the LRRK2‐G2019S average values to 0 and LRRK2‐WT to 100%. We then evaluated the effect of Inh2 to restore mutant lines toward a LRRK2‐WT status after 6w of neuronal differentiation. Inh2 rescued TH‐related features (TH^+^, skeleton TH, links, and nodes) of LRRK2‐G2019S lines. The rescue size effect was: 82.6% for TH^+^, 64.4% for skeleton TH, 43% for links, and 48.4% for nodes (Figure 3C). The same analysis was performed in patient lines genetically corrected to LRRK2‐WT. We evaluated the rescue effects of gene correction by setting the LRRK2‐G2019S average values to 0% and the LRRK2‐WT line to 100%. As for the pharmacological treatment, gene editing also resulted in a phenotype amelioration, but at a lower amplitude. The rescue size effect was: 19.2% for TH^+^, 30.1% for skeleton TH, 39.2% for links, and 41.1% for nodes.

### 2.4 Contribution of Patient‐Specific Genetic Background to the LRRK2‐G2019S Phenotype

**Figure 4:**
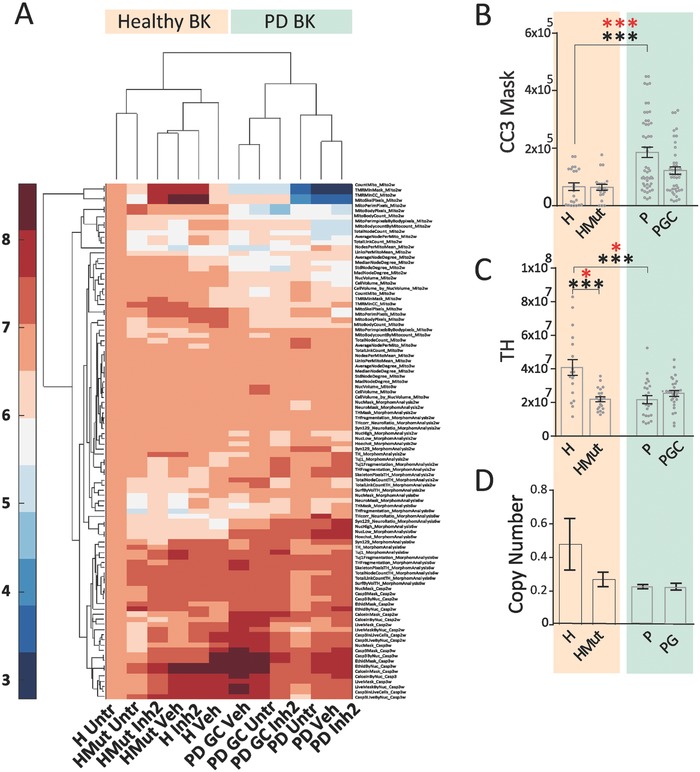
Contribution of genetic background to LRRK2‐G2019S‐induced neuronal abnormalities. A) Heatmap comprising of all the image analysis assays combined, showing a clear clustering of lines according to genetic background (BK). Bar graphs showing B) CC3 Mask after 2w (number of bioreactors: H 23, H Mut 21, P 51, PGC 45); C) TH pixels after 6w (number of bioreactors: H 18, H Mut 21, P 23, PGC 28); and D) mitochondrial copy number after 3w (the experiment was performed five times, in triplicates, with all the lines in Figure S1A (Supporting Information); the technical replicates were averaged). Bars represent means ± SEM. In (B) and (C) each dot represents a bioreactor. *p*‐values are calculated using Kruskal–Wallis and Dunn's post hoc test. They are adjusted (red) or not (black) according to Benjamini–Hochberg (number of total features assed 9 for (B) and 18 for (C), *p ≤ 0.05, **p ≤ 0.01, ***p ≤ 0.001. Dunn's post hoc analysis was performed on the following pairs: H/HMut, H/P, P/PG. H = healthy lines, HMut = healthy lines where LRRK2‐G2019S mutation was introduced, P = PD lines carrying LRRK2‐G2019S mutation, PGC = PD lines where LRRK2‐G2019S mutation was corrected. In (A)–(C), 420 fields at 20× magnification were acquired for each bioreactor (21 fields for 20 planes).

We next stratified the lines for the genetic background of the donors (PD patients or healthy individuals). The clustergram shows that PD patient‐derived lines cluster together independently of the presence or absence of the mutation (**Figure** **4**A). This suggests that the genetic background of cells accounts for most of the differences between the studied cell lines, regardless of pharmacological treatment or gene editing. A closer analysis of some of the underlying assays showed that PD patient‐derived lines (P) presented significantly higher levels of CC3 in live cells compared to lines derived from healthy individuals (H) (Figure 4B). LRRK2‐G2019S correction to LRRK2‐WT sequence (PGC) rescued CC3 activation without restoring the levels back to H. The TH pixel count was reduced in both the inserted mutation (HMut) and P (Figure 4C). We also quantified mtDNA copy number in neurons as indicators of mtDNA maintenance and observed a similar pattern. When comparing H and P neurons, the latter showed a trend toward reduced copy number and transcription/replication rates. Insertion of the LRRK2‐G2019S mutation in the healthy line (HMut) mirrored this phenotype. By contrast, LRRK2‐G2019S correction in the patient line (PGC) did not lead to an increase in mtDNA copy number comparable to LRRK2‐WT levels (Figure 4D).

### 2.5 Assay Performance Based on Receiver Operating Characteristic (ROC) Analysis

To estimate the discriminative performance of single assays using ROC curve analysis, we performed the following comparisons: LRRK2‐G2019S versus LRRK2‐WT (Figure S4A, Supporting Information), and LRRK2‐G2019S Inh2 versus LRRK2‐WT Inh2 (Figure S4B, Supporting Information). Table S1 (Supporting Information) indicates the number of samples for each group, as well as the number of features extracted for each assay. For cell death at 2w, and mitochondrial assays at 2w and 3w, we observed an acceptable trade‐off between number of samples and features. Cell death at 3w or morphometric analysis at 2w and 6w did not reveal similar effects. In the morphometric assay, a large number of features was extracted. In these three cases, we applied the feature‐selection approach as described in the Experimental Section: the chosen threshold was the entity yielding the best results among the values 0.75, 0.8, 0.85, and 0.9. Except for the comparison of the morphometric assay at 6w between LRRK2‐G2019S Inh2 and LRRK2‐WT Inh2, where the threshold was 0.75, the chosen threshold was 0.8 for all the other cases. **Table** **2** shows the results for the comparison between LRRK2‐G2019S and LRRK2‐WT. The cell death assays produced a greater separation between the two conditions, as they provided an area under the curve (AUC) of 88.5 for 2w and 90.1 for 3w with high accuracies, sensitivities, and specificities. The morphometric assays provided reasonable performances (77.9 at 2w and 79.2 at 3w). Finally, the mitochondrial assays showed low performance in discriminating the two conditions despite providing good values of specificity. Additionally, **Table** **3** shows the results for the comparison between LRRK2‐G2019S Inh2 and LRRK2‐WT Inh2. The cell death assays maintained the highest AUCs, though with lower values compared to Table 2. For the remaining assays, the AUCs were lower compared to Table 2, indicating that addition of Inh2 brought LRRK2‐G2019S values closer to LRRK2‐WT.

**Table 2:** Results for the comparison between LRRK2‐G2019S and WT

Assay and time point	Features after selection	AUC [mean ± std]	Accuracy [%]	Sensitivity [%]	Specificity [%]
Cell death 2w	11	88.5 ± 2.2	81	81.9	80.1
Cell death 3w	6	90.1 ± 3.9	85.8	81.6	89.3
Mitochondrial assay 2w	21	63.4 ± 4	59.8	46.5	71.5
Mitochondrial assay 3w	21	56.5 ± 4.9	54.9	39.4	69.1
Morphometric analysis 2w	16	77.9 ± 3.3	70.6	70.4	70.8
Morphometric analysis 6w	16	79.2 ± 3.5	75.1	63.1	84.5

**Table 3:** Results for the comparison between LRRK2‐G2019S Inh2 and LRRK2‐WT Inh2

Assay and time point	Features after selection	AUC [mean ± std.]	Accuracy [%]	Sensitivity [%]	Specificity [%]
Cell death 2w	11	81.1 ± 2.4	67.9	66.1	70
Cell death 3w	6	74.1 ± 4.1	67.5	67	68
Mitochondrial assay 2w	21	71.7 ± 3.1	66.7	84.6	46.2
Mitochondrial assay 3w	21	65.1 ± 3.7	62.2	83.8	36.1
Morphometric analysis 2w	20	52.2 ± 5.7	54.1	54.7	53.6
Morphometric analysis 6w	14	67.7 ± 4.1	62.4	80.5	38.5

## 3 Discussion

Recapitulating the key cellular hallmarks of LRRK2‐associated toxicity in patient‐derived cells is a prerequirement to set up in vitro assays, which can drive personalized medicine approaches. Here, we developed a platform based on hNESC‐derived neurons from PD patients carrying the LRRK2‐G2019S mutation. This platform recapitulates key features of PD, including degeneration of dopaminergic neurons and preceding mitochondrial impairments. Treatment with the LRRK2‐specific inhibitor, Inh2, rescued neurodegeneration, and neurite complexity phenotypes without fully reversing mitochondrial abnormalities. When considering all experiments at all time points, genetic background of the patients was found to be a major discriminating factor among the lines and not the LRRK2‐G2019S mutation.

Cells in a 3D in vitro setting are subjected to structural cues, which bring them closer to physiological conditions.^23^ This confined microenvironment seems to leverage cell–cell contact and extracellular matrix protein synthesis.^24^ Unlike in 2D cultures, cells grown in 3D microenvironments exhibit gene expression patterns and cellular phenotypes that resemble in vivo conditions.^25^ This metabolic‐competent system, combined with stem cell technology tools, holds the potential to leverage the assessment of drug effects to a human‐derived model at a very early stage. Classical in vitro models for PD are mainly comprised of PD‐patient fibroblasts or immortalized cells (e.g., SH‐SY5Y) with all the associated drawbacks, including different gene expression, compared to neurons.^26^ The successful use of 3D‐derived and hNESC‐derived neurons for phenotype assessment has been demonstrated with Alzheimer's disease.^9^ As paracrine factors diffuse rapidly into large media volumes in 2D cultures, we hypothesized that neurons cultivated in 3D exacerbated LRRK2‐G2019S toxicity by providing a brain tissue‐like environment. We chose Matrigel as a 3D support matrix, as it contains high levels of brain extracellular matrix proteins such as laminin, collagen, and heparin sulfate proteoglycans.^27^ The 3D scaffold given by Matrigel, but also other hydrogels such as collagen and alginate gels, has shown to accelerate neuronal network formation.^9^, ^28^ More importantly, the support of the matrix permits vertical growth, which is completely lacking in 2D cultures and results in unwanted apical‐basal polarity.^4^ The 3D environment is also able to trigger mechanical cues which can be converted into biochemical signals not achievable in conventional flat cultures. Building on this, several brain organoids recapitulating cortical^29^ and midbrain^30^ identities have recently been developed and demonstrated the huge potential of 3D systems to recapitulate disease‐relevant features. The derivation of dopaminergic neurons in the described 3D set‐up also has the advantage of using low volumes of media and the option for multiplexing and automated screening activities.^31^ Beyond enabling larger throughput than manual approaches, the automation also improves reproducibility and the depth of available phenomic data, allowing a systems‐level understanding of the multiple contributions of LRRK2 in determining neuronal phenotypes. A deeper understanding of the role of LRRK2 in PD is of interest, as the same pathways may be shared with idiopathic PD.^32^

Our results are consistent with a series of past observations describing reduced speed of neurite outgrowth in LRRK2‐G2019S‐mutant neurons within 30 min of recording in bright field microscopy.^33^ LRRK2‐G2019S or Y1699C‐LRRK2 expression led to shorter total neurite lengths compared to wild‐type rat primary neurons.^34^ However, in contrast to previous reports, here we were able to show a progressive time‐dependent degeneration of TH^+^ neurons carrying LRRK2‐G2019S. Interestingly, at 2w, we observed an increased TH/Tuj1 ratio, which we hypothesize is an attempt to accelerate dopaminergic differentiation to counteract LRRK2‐induced defects. This compensatory mechanism has often been described in other pathological contexts, for instance, in Alzheimer's disease brain samples, where the synaptic protein synaptophysin is increased in autoptic brain stages 3 and 4 and later decreased in Braak stage 5–6.^35^

The hypothesis of mitochondrial dysfunction playing a major role in PD pathogenesis has been proposed in several studies.[[qv: 19c,36]] Interestingly, LRRK2 seems to control mitochondrial homeostasis via the dynamin‐like protein DLP1.^37^ We have confirmed compromised mitochondrial function in LRRK2‐G2019S cells,^20^, ^37^, ^38^ but also broadened its characterization in hNESC‐derived neurons. It has been reported that overexpression of LRRK2‐G2019S in SH‐SY5Y cells causes reduced membrane potential without changes in mitochondrial morphology.^20^ By contrast, 2D‐derived LRRK2‐G2019S iPSC‐derived neurons exhibited an intact electron transport chain, yet susceptibility to stress after chemical stressors was evident.^39^ These differences can be reconciled in our model, where no tumoral lines were used and no chemical stressors were applied. The 3D condition was the only factor triggering a striking alteration in mitochondrial number and network complexity. LRRK2 controls microtubule stability, which is essential for the trafficking of mitochondria to every distal area in branched cells. LRRK2‐G2019S was shown to increase phosphorylation of tubulin, thereby altering the microtubule dynamics^40^ and destabilizing the mitochondrial network as well.

In contrast to previous studies,^41^ we also showed the occurrence of neuronal cell death without using neurotoxins. Only a modest increase in CC3 was previously observed in 2D cultures even after treatments with H_2_O_2_ or 6‐OHDA.[[qv: 41a]] The increased CC3 levels and LDH release observed here highlight the intrinsic vulnerability of neurons carrying LRRK2‐G2019S mutation. A reason for increased cell death in LRRK2‐G2019S neurons may lie in its interaction with 14‐3‐3s, a family of proteins that plays a role in cell survival.^42^ Mutations of the residues S910/S935 to alanine in LRRK2 decreased its interaction with 14‐3‐3, causing toxic cellular redistribution of LRRK2 in HEK293 cells.^42^

Due to its involvement in familial and sporadic PD and the presence of a drugable kinase domain, LRRK2 has become an attractive pharmacological target. Initial studies focused on LRRK2 kinase activity as a pathological trigger.^43^ Subsequent investigations also pointed to protein expression levels, focusing on posttranslational modifications such as phosphorylation at serine residues Ser910 and Ser935, located prior to the leucine‐rich domain of LRRK2.[[qv: 43b]] With a view on therapeutical application, we used Inh2 as a proof of concept to gain some mechanistic insights. In our set‐up, Inh2 rescued dopaminergic expression but not mitochondrial and viability abnormalities. The fact that LRRK2‐G2019S, the most active mutation in increasing kinase activity,[[qv: 43b]] is not fully penetrant suggests that its pathological function comprises of additional pathways. Permissive genetic background, due to cumulative genetic variants, might mediate and enhance LRRK2‐induced neurodegeneration. From this perspective, proper patient stratification is essential for the identification of therapeutic choices with maximized effect probability. For example, trafficking of dopamine receptor 1 and 2 seems to be affected by LRRK2‐G2019S leading to alterations in signal transduction.^44^ This might result in resistance to the neurotrophic effect of dopamine receptor antagonists that could be preliminary tested for therapy prioritization. The possibility of using 3D in vitro testing to stratify PD patients for proper drug administration is a key opportunity to bring the present work to clinical application.

Despite its advantages, 3D microfluidic systems still present several limitations including proper control of critical factors, for example, oxygen tension, pH, and gradient‐dependent effects within the matrix. Thus, the applicability for drug screening purposes is currently still a proof‐of‐concept, which needs to be further validated. Moreover, in our current set‐up, only neurons were assessed, yet other cell types may be affected and contribute to PD pathology.^45^ Cocultures of various iPSC‐derived neural cells will be important to assess cell and noncell autonomous effects. hNESC‐derived neurons successfully recapitulate hallmarks of LRRK2 pathogenesis including degeneration, cell loss, and mitochondrial impairment. However, we did not detect α‐synuclein accumulation as previously observed.^33^ The improved microenvironment seems insufficient in overcoming the limitation of mirroring robust α‐synuclein accumulation within 6w. A major challenge is to comprehensively recapitulate PD pathology in vitro over a short time‐course, whereas in vivo, this takes decades. A more thorough recapitulation might come with the administration of aging‐associated stressors such as DNA damaging agents or oxidative stress triggers.^46^ Further studies are necessary in this aspect and will certainly fuel our understanding of disease pathogenesis and options for drug discovery.

## 4 Experimental Section

*Cell Lines*: In this paper, the cell lines used for all the experiments, unless otherwise indicated, are described in Figure S1A (Supporting Information). Two lines were obtained from two healthy individuals and two lines from two PD patients carrying the LRRK2‐G2019S mutation. To create the isogenic lines, the mutation was either corrected or introduced. From Figures 1 to 3 and in the Supporting Information, the grouping for the lines was done according to the LRRK2 sequence (LRRK2‐WT or LRRK2‐G2019S), independently of the genetic background (healthy or PD). Only in Figure 4, the genetic background of the lines was taken into account. In Figure S3A,B (Supporting Information), the microarray analysis was performed in hNESC from six healthy individuals and six PD patients carrying LRRK2‐G2019S.

The hiPSC‐derived hNESCs were generated and cultured as previously described.^16^ For the Healthy 2 line, the footprint‐free isogenic LRRK2‐G2019S cell pair was established using CRISPR/Cas9 and piggyBac.^47^ Besides the p.G2019S (c.G6055A) mutation inserted into the wild type LRRK2 locus, a silent mutation (c.A6087T) was also introduced to convert TAAA into TTAA that is required for piggyBac insertion and release. To make the wild type control an authentically isogenic control, the silent mutation (c.A6087T) was introduced into wild type hiPSC as well, but without the p.G2019S mutation. The described LRRK2‐G2019S isogenic cells differed only by LRRK2‐G2019S from the Healthy2 line and the pair underwent the same procedure of editing and subcloning.

For cultivation, the hNESC derived from iPSC of two PD patients and two healthy individuals and their isogenic controls were cultured in N2B27 medium: Dulbecco's modified Eagle's medium‐F12 (Gibco)/Neurobasal (Gibco) 50:50 supplemented with 1:200 N2 (Invitrogen), 1:100 B27 lacking vitamin A (Invitrogen), penicillin/streptomycin, and glutamine (Invitrogen). 3 × 10^−6^ m CHIR 99 021 (Axon), 0.5 × 10^−6^ m purmorphamin (PMA), (Enzo Life Science), and 150 × 10^−6^ m ascorbic acid (Sigma) were added. Cells were maintained in Matrigel‐coated plates. At a confluence of 70–80%, cells were detached using Accutase (Life Technologies) for 3 min, collected by centrifugation and resuspended in 80% Matrigel (BD Bioscience). 27 000 cells were loaded in microfluidic OrganoPlates (Mimetas) in 0.8 µL per bioreactor. The media perfusion was achieved by gravity, with an average fluid flow of 1.5 µL h^−1^.

To achieve neuronal differentiation, cells were cultured in N2B27 medium supplemented with 10 ng mL^−1^ brain‐derived neurotrophic factor (Peprotech), 10 ng mL^−1^ glial cell‐derived neurotrophic factor (Peprotech), 1 ng mL^−1^ transforming growth factor‐β3 (Peprotech), 200 × 10^−6^ m ascorbic acid, and 500 × 10^−6^ m dibutyryl cyclic‐AMP (Sigma‐Aldrich). For the first 6 days, 1 × 10^−6^ m PMA was also added and the media was changed every second day. From day seven onward, media without PMA was changed every fourth day (Figure 1A).

For 2D neuronal differentiation, 10 000 cells per well were seeded on Matrigel‐coated 96 well cell carrier plates (PerkinElmer). The same differentiation protocol used in the OrganoPlates was applied.

For drug treatments, differentiation media containing 0.5 × 10^−6^ m LRRK2 Inh 2 (Merck Chemicals, CZC‐25 146) or dimethyl sulfoxide (DMSO) (Sigma) as vehicle were added at every media change.^22^ The final DMSO concentration in the media was 0.1% for Inh2 and vehicle. In this paper, cells treated with DMSO were labelled as LRRK2‐G2019S or LRRK2‐WT.

*Microarray Generation and Analysis*: RNA was extracted from six healthy and six PD LRRK2‐G2019S patient‐hNESC lines using miRNA easy kit following the manufacturer's instructions (Qiagen). Samples were processed with EMBL Genomics Core Facility using Affymetrix Human Gene 2.0 arrays. Differential expression was analyzed in the R statistical programming framework (R Development Core Team 2011) using the RankProduct method (Breitling et al., 2004). The data are accessible under the GEO accession number GSE101534. The Heat map shows 29 of 865 differentially expressed genes between healthy individuals and patients that overlap within gene lists from the Human MitoCarta2.0.

*Mitochondria Live Imaging*: Mitochondrial membrane potential (ΔΨ) was assessed with TMRM (Thermo Scientific). Neurons differentiated for 2w and 3w in OrganoPlates were subjected to TMRM (4 × 10^−9^ m) to analyze for their mitochondrial membrane potential, along with MitoTracker (1:10 000) Green (Invitrogen). In addition, cells were costained with Hoechst (1:1000) and Cell Mask (1:5000) to visualize nuclei and cell bodies, respectively (Invitrogen). Cells were incubated for 30 min at 37 °C. Fluorescence images were acquired on Opera confocal microscope (PerkinElmer).

*Immunofluorescence Staining*: In both 3D and 2D groups, neuronal cultures were fixed with 4% paraformaldehyde (PFA) in 1x phosphate buffer (PBS) overnight at 4 °C. After 3 washes in PBS, cells were permeabilized for 15 min in 0.3% Triton‐X100 in PBS at RT. After blocking for 1 h (2% fetal bovine serum, 2% bovine serum albumin, 0.1% Triton‐X100), the first antibodies were incubated overnight at room temperature. A combination of the appropriate secondary antibodies (Invitrogen) was then added for an additional 2 h at room temperature. Cells were analyzed by the neuronal marker Tuj1 (Millipore), the dopaminergic marker TH (Santa Cruz Biotechnology), and the dye Hoechst. The semiquantitative expression of LRRK2 phosphorylated at the serine 935 (pS935) (Abcam) and α‐synuclein phosphorylated at the serine 129 (pS129) (Abcam) was assessed.

To evaluate cell viability in 3D, calcein and ethidium homodimer costaining was used (Life Technology). Neurons were incubated for 45 min and subsequently fixed with 4% PFA overnight at 4 °C to perform CC3 (Cell Signaling Technology). Fluorescence images were acquired on Opera confocal microscope (Perkin Elmer) with a 20× Objective.

*Image Analysis of Morphometric Assays*: Immunofluorescence four channel 3D images of hNESC derived neuronal cultures in OrganoPlates were analyzed in Matlab (2017a, Mathworks). The developed custom image‐analysis algorithm automates four major steps, namely: mosaic stitching, segmentation of bioreactors, segmentation of nuclei and neurons, and feature extraction.

For stitching, normalized cross correlations between overlapping image sections were computed and positions of the local maxima were used to return x and y offsets. Positioning of images in the mosaic was implemented accordingly, using translation.

Bioreactors were segmented based on fluorescence intensities in Hoechst, Alexa488, Alexa568, and Alexa647 channels. For preprocessing, each channel was average filtered with a square‐shaped structuring element of side length 5 and maximum projection. A first rough bioreactor segmentation to refine the bioreactor mask was used to remove false‐positive pixels and to leverage the detection of vertical linear structures in the microfluidic device. To remove sparse cellular structures, the rough bioreactor mask was eroded with a disk‐shaped structuring element of radius 5. Connected components with less than 1000 pixels were removed. To enlarge the bioreactor mask, dilatation with a disk‐shaped structuring element of radius 20 was applied. To close vertical gaps, the mask was dilated with a vertical rod‐shaped structuring element with a height of 101 and width 1. To remove potentially remaining false‐positive structures in microfluidic channels, erosion with a vertical rod‐shaped structuring element of height 501 and width 1 was applied. Clipped objects with less than 100 000 pixels were removed. To detect the phase guide, connected components were identified using the function bwconncomp—resulting in three blocks in an increasing manner from left to right. The middle block with index 2 was defined as phase guide. Next, the 2D‐ bioreactor mask was projected to all planes of the 3D‐bioreactor mask and the phaseguide was clipped to the lower eight planes. Segmentation of the Matrigel channel (MC) and perfusion channel (PC) was based on the fact that the MC is located to the left of the phaseguide while the PC is located on the right side. The tool used for this step is the Matlab function imreconstruct, which reconstructs objects within a limiting mask, provided that they contain corresponding pixels in a seed mask. The limiting mask in both cases was the complement of the bioreactor mask. For reconstruction of the MC, the seed mask was created by dilating the phaseguide mask with the structuring element [1 1 0]. Similarly, dilatation with the structuring element [0 1 1] was used to create a seed mask for the reconstruction of the PC.

After segmenting the bioreactor, nuclei and neurons were also segmented. Image preprocessing for segmentation of nuclei was calculated via difference of Gaussians. Briefly, a foreground image was computed by convolving the raw Hoechst channel with a Gaussian filter of size 10 and standard deviation 2. Similarly, for background images, a Gaussian filter of size 60 and standard deviation 20 was used. The difference was computed by substracting the background from the foreground. The first rough‐nuclei mask was defined by pixels with gray tone values larger than 10. To refine the nuclei mask, pixels overlapping with the OrganoPlate mask were removed and only connected components with at least 200 pixels were retained. To classify nuclei pixels based on fluorescence intensity, the raw Hoechst channel was preprocessed via average filtering with a square‐shaped structuring element of side length 5. Pixels overlapping with the nuclei mask and with values above 400 were assigned to the pyknotic‐nuclei mask. The remaining nuclei mask pixels were assigned to the normal nuclei mask.

For the segmentation of neurons, a strategy combining global and local thresholding was implemented. For global thresholding, image preprocessing was performed via low pass filtering. For this purpose, the raw Tuj1 channel was convolved with a Gaussian filter of size 10 and standard deviation 3. The global neuronal mask is defined by threshold 150. For local thresholding, a difference of Gaussians was applied in the preprocessing step. Precisely, the background defined via convolution with a Gaussian filter of size 20 and standard deviation 6 was substracted from the foreground defined via convolution with a Gaussian filter of size 10 and standard deviation 3. The local neuronal mask is defined by those pixels with values larger than 3. The concepts of global and local thresholding were combined by retaining those pixels in the neuronal mask, which were detected by at least one of these methods. For refining the neuronal mask, connected components with less than 200 pixels were removed.

To analyze neuronal fragmentation, the concept of erosion was used. Indeed, the surface of fragmented objects is larger than the surface of nonfragmented objects as compared to their cumulated volumes. For the analysis of fragmentation in the TH channel, an additional mask was defined by preprocessing the raw TH channel via convolution with a Gaussian filter of size 10 and standard deviation 1, and thresholding of this image by pixel value 100. Both masks were eroded with a 3D‐structuring element corresponding to a center pixel and its 6‐connected neighborhood. Furthermore, the surface masks were computed by subtracting eroded masks from corresponding original masks.

Some of the extracted features are summarized in Table 1.

*Image Analysis for the Mitochondrial Assays*: The custom image analysis algorithm developed for the processing of TMRM assays automates the same key steps as above. Since a different staining was used as compared to the morphometric assays, the first raw segmentation of the OrganoPlates was based on the rule OrganoPlate Mask = 10 < TMRM_max_ < 30 or 30 < Hoechst_max_ < 200 and MitoTrackerGreenFM_max_ > 15, where subscript max refers to maximum projections of size 5 average filtered raw images. The refinement of the OrganoPlate mask was done as described above.

The nucleus mask was defined by global thresholding (>100) in the raw Hoechst channel. Pixels which were not included in the OrganoPlate mask were removed. The CellMask was defined via a combination of local and global thresholding. Large and bright cellular structures were identified via thresholding on the raw CellMask channel (>400). Smaller cellular structures were detected via local thresholding by applying a difference of Gaussians to the raw CellMask channel. The foreground image was computed via Gaussian convolution with size 100 and standard deviation 5. The subtracted background image was defined via Gaussian convolution of size 100 and standard deviation 30. The cell mask was defined via thresholding of this difference of Gaussians (>10) and by removing pixels assigned to the OrganoPlate mask. The mitochondrial mask was defined via a difference of Gaussians. For the foreground image, the Gaussian size was set to 10 and the standard deviation to 1. For the subtracted background, a Gaussian of size 10 and standard deviation 3 were used. A first raw mitochondria mask was defined via thresholding of this difference of Gaussians (>30). To refine the mitochondria mask, connected components with less than 5 or more than 500 pixels, and the OrganoPlate mask were removed.

In order to leverage the morphometric analysis of mitochondria, the surface of mitochondrial‐connected components and the corresponding mitochondrial bodies were defined via erosion of the mitochondria mask with a 3D‐structuring element corresponding to a pixel and its 6‐connected neighborhood. The skeletonization of the mitochondrial mask was performed using established methods.^48^ The extracted features are summarized in Table 1.

*Image Analysis for the Cell Death Assays*: The custom image analysis algorithm developed for the analysis of Live/Dead assays automates the same key steps as above. The first raw segmentation of OrganoPlate bioreactors was based on the rule OrganoPlate Mask = 50 < Calcein_max_ < 300, where subscript max refers to the maximum projection of size 5 average filtered raw Calcein images. For the refinement of the OrganoPlate mask the size of the structuring element used for closing vertical gaps was set to height 201 and width 1 and the size of the structuring element used for the subsequent erosion was set to height 3001 and width 1. In addition, the mask was opened with the function imopen, using a disk‐shaped structuring element of radius 20 and dilated using a disk‐shaped structuring element of radius 22.

Segmentation of nuclei was based on global thresholding on a difference of Gaussians of the Hoechst channel. The foreground image of the difference of Gaussians was computed via a Gaussian kernel of size 10 and standard deviation 2. The subtracted background image was returned from Gaussian convolution using size 60 and standard deviation 20. The nuclei mask was defined via thresholding (>50). Next, the nuclei mask was refined by excluding pixels from the OrganoPlate mask and by removing connected components with less than 200 pixels. To define the mask of EH‐positive pixels, the corresponding channel was low‐pass filtered using a Gaussian filter of size 10 and standard deviation 3. Next, thresholding was applied (>500) and pixels overlapping with the OrganoPlate were excluded. For detecting CC3 positive pixels, the raw CC3 channel was low‐pass filtered with a Gaussian of size 10 and standard deviation 3. The threshold was set to 250 and in the resulting mask, pixels overlapping with the OrganoPlate or nucleus masks were excluded. Furthermore, connected components with less than 20 pixels were removed. For the detection of Calcein‐positive pixels, an approach combining global and local thesholding was used. For global thresholding, the raw Calcein channel was low‐pass filtered with a Gaussian of size 10 and standard deviation 1, and the threshold was set to 50. For local thesholding, a difference of Gaussians was computed on the Calcein channel. The foreground Gaussian was set to size 20 and standard deviation 1. The background Gaussian was set to size 20 and standard deviation 5. The threshold was set to 10. The local and global Calcein masks were combined via Boolean OR operation and pixels overlapping with the nuclei mask or the OrganoPlate mask were removed from the Calcein mask. The live mask was defined by excluding the nuclei mask from the Calcein mask and by removing connected components with less than 200 pixels. The CC3 live mask was defined via Boolean operations using the Calcein mask, the not nuclei mask, and the CC3 mask. Extracted features are shown in Table 1.

*LDH Viability Test*: Cell death was assessed in 3D microfluidic devices at 3 weeks of neuronal differentiation by measuring the amount of LDH released into cell culture media by plasma membrane‐damaged cells. The assay was conducted following manufacturer's instructions (Pierce LDH Cytotoxicity Assay kit, Thermo Scientific). Briefly, the absorbance was measured at 490 and 680 nm (background signal from the instrument). To calculate % of cytotoxicity, for each experiment the LDH activity of the spontaneous release control (water‐treated) was subtracted from the LDH activity of the lysis buffer‐treated samples and multiply by 100 in the case of the LRRK2‐WT cells. The increase in the release of LDH in LRRK2‐G2019S was expressed as % of LRRK2‐WT. The experiment was performed in quadruplicate, nine times in LRRK2‐WT and LRRK2‐G2019S lines. Technical replicates were averaged.

*Zn Concentration Determination*: Zn concentration was evaluated in the media collected in the outlet well of the bioreactors over the course of the neuronal differentiation. 200 µL of medium outflow was collected into 2 mL polypropylene vials (Nalgene cryogenic vials, Merck) for each condition at 3 different time points (1st, 3rd, and 6th week). 20 µL of nitric acid (HNO_3_) 65% were added for sample stabilization. Prior instrumental analysis, these samples were digested at 60 °C for 48 h in 200 µL of 1 + 1 mixture of 30% H_2_O_2_ s.p. and 65% HNO_3_ s.p. (Merck, Darmstadt, Germany). The obtained clear solutions were then diluted with milliQ water up to 2 mL. Concentrations of Zn in digested media outflow samples were determined by inductively coupled plasma mass spectrometry (Agilent 7900 series, Tokyo, Japan) under the following operating parameters: forward power 1550W, plasma gas flow 15.0 L min^−1^, carrier gas flow 0.95 L min^−1^, dilution gas flow 0.1 L min^−1^, sample depth 8.0 mm, He gas flow 4.5 mL min^−1^, and energy discrimination 5 V. The isotope monitored was Zn^66^ and the isotopes of internal standards ^103^Rh and ^193^Ir. Because of the sample preparation, the Zn levels were assessed independently from the metal oxidation state. Time‐dependent quantification of Zn was expressed as ng mL^−1^. The experiment was performed three times and the media of four technical replicates was pulled each time. Healthy1 and Healthy1‐Mut lines (see panel Figure S1A, Supporting Information) were used.

*Mitochondrial Genome Analysis*: In order to collect sufficient material, this analysis was performed in 3D thick cultures embedded in Matrigel in a 24‐well plate with inserts as previously described.^9^ 1 million hNESC were seeded inside the inserts in 50% Matrigel. mtDNA copy number analysis was performed according to a previously published protocol using TagMan probes (Applied Biosystems) targeting MT‐ND1 and the nuclear gene B2M.^49^ In addition, the multiplex real‐time PCR assay was employed to quantify 7S DNA relative to MT‐ND1.^50^ 7S DNA is a DNA strand that is incorporated in the D‐Loop region during transcription and replication of the mitochondrial genome and can therefore serve as an indicator of “active” mtDNA molecules.^51^ The experiment was performed five times, in triplicates, with all the lines in Figure S1A (Supporting Information). The technical replicates were averaged.

*Multivariate Classification Methods*: To study the potential of the described assays in robustly discriminating the different groups, multivariate classification methods were used. The performance was first measured in terms of accuracy in classification, sensitivity, and specificity. However, since the dataset includes different numbers of samples per group, a ROC analysis was additionally performed, which measures the true‐positive rate (sensitivity) against the false‐positive rate (specificity). From the generated ROC curves, the area under the ROC curve (AUC) was computed. AUC is a reliable measure of performance, which takes the skewness in the sample distribution into account. For the binary classification between the groups of interest, support vector machines were applied with a radial basis function (RBF) kernel to boost accuracy. The results were evaluated using a fivefold cross validation. We randomly split the set of LRRK2‐G2019S and LRRK2‐WT samples in two subsets: a training set that comprises 4/5 of the samples, and a test subset with the remaining 1/5 samples. Then, the classifier was trained using the training set, and its accuracy was estimated by testing its performance on the test set. This was run for the five different combinations of training and test sets. This process was repeated 200 times to ensure statistical robustness for both accuracy and AUC estimation. We also address the tendency of the RBF kernel to overfit during the training process. Hence, we additionally trained the classifier using a wide range of values of the two parameters the classifier depends on. For the parameter that controls the overfitting, we generated 21 values ranging from 10^−5^ to 10^5^, and for the parameters that scales the RBF kernel, we produced ten values between 10^−3^ and 10. Equal spacing was ensured logarithmically. For the two comparisons presented in the current paper (LRRK2‐G2019S vs LRRK2‐WT and LRRK2‐G2019S Inh2 vs LRRK2‐WT Inh2), all considerations previously stated helped ensure the validity of the obtained results for cell death and mitochondrial assays at their two time points, since there was a fair trade‐off between number of samples and number of features. Nevertheless, for the morphometric analysis assay, the number of samples for each comparison was insufficient compared to the number of features, which might have led to strong overfitting. Therefore, we decided to additionally proceed with a stage of feature selection before the classification. We took advantage of the existing high correlation between some of the designed features in the morphometric assay by removing one of each pair of highly correlated features at a time. We repeated this process iteratively until the remaining features produced cross‐correlations below the given threshold. For the generation of the results, we used Matlab (R2017a).

*Statistics*: In all the image analysis data, every data point corresponds to the analysis of the cells in one entire bioreactor chamber. The overall number of bioreactors analyzed is indicated in the figure legend. The data extracted from the image analysis was not normalized but it was kept as a raw value. Outliers were removed using Matlab according to the interquartile rule where an outlier is bigger than the third quartile plus the interquartile range (1 × IQR) or smaller than the first quartile minus 1 × IQR. The statistical analysis was performed in R using either Mann Whitney test when 2 conditions were compared or Kruskal–Wallis when more than 2 conditions were compared. Dunn's post hoc test was performed on selected comparisons indicated in the figure legends. Adjustment of p‐values was performed using Benjamini–Hochberg method based on the number of features analyzed and on the number of comparisons performed. Graphs were generated in GraphPad Prism software. Levels of significance are as follows: *p ≤ 0.05, **p ≤ 0.01, ***p ≤ 0.001. α value was for all statistics 0.05. Data in dot plots are expressed as mean values ± S.E.M. In all box‐plots the whiskers represent the minimum and maximum values of the distribution. To analyze the rescue size effect given by Inh2 or gene editing, a bootstrap method with 1 × 10^5^ iterations and ten randomly chosen samples per iteration was used.

## Conflict of Interest

The authors declare no conflict of interest.

## Supporting information

Supplementary
