# Supplementary material for: 3D Cultures of Parkinson's Disease‐Specific Dopaminergic Neurons for High Content Phenotyping and Drug Testing
Source: Adv Sci (Weinh). 2018 Nov 20;6(1):1800927. doi: 10.1002/advs.201800927 (PMC6325628; doi:10.1002/advs.201800927)
Supplement: Supplementary file 1 — Supplementary [file ADVS-6-1800927-s001.pdf]

Copyright WILEY-VCH Verlag GmbH & Co. KGaA, 69469 Weinheim, Germany, 2018.

Supporting Information

for *Adv. Sci.,* DOI: 10.1002/advs.201800927

3D Cultures of Parkinson's Disease-Specific Dopaminergic Neurons for High Content Phenotyping and Drug Testing

Silvia Bolognin, Marie Fossépré, Xiaobing Qing, Javier Jarazo, Janez Ščančar, Edinson Lucumi Moreno, Sarah L. Nickels, Kobi Wasner, Nassima Ouzren, Jonas Walter, Anne Grünewald, Enrico Glaab, Luis Salamanca, Ronan M. T. Fleming, Paul M. A. Antony, and Jens C. Schwamborn**

A

Line	Age at sampling	Gender	Reference	LRRK2
Healthy1	81	F	Reinhardt et al., 2013	WT
Healthy1-Mut	81	F	Reinhardt et al., 2013	G2019S
Healthy2	55-59	M	Qing et al., 2017	WT
Healthy2-Mut	55-59	M	Qing et al., 2017	G2019S
PD1	81	F	Reinhardt et al., 2013	G2019S
PD1-GC	81	F	Reinhardt et al., 2013	WT
PD2	54	F	Reinhardt et al., 2013	G2019S
PD2-GC	54	F	Reinhardt et al., 2013	WT

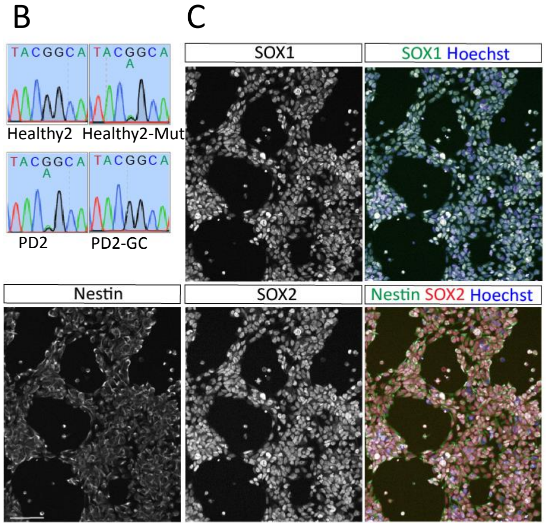

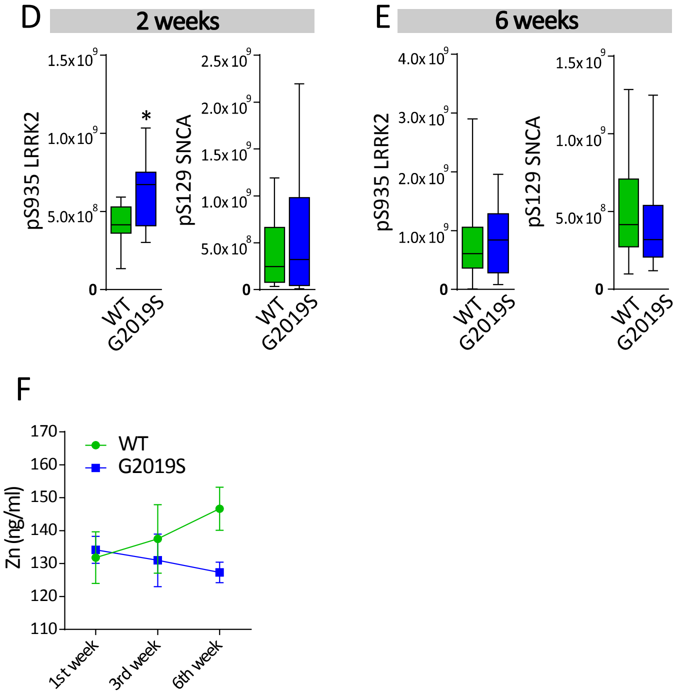

Fig. S1. Quality controls of hNESC and 2D neurons. (**A**) Table illustrating the characteristics of the cell lines used in the study. (**B**) Genomic sequencing confirming the insertion of LRRK2-G2019S mutation in healthy cell lines, and correction of the mutation in PD cell lines. (**C**) Representative confocal pictures of hNESC stained against stem cell markers SOX1, SOX2, and Nestin as a quality control of the cultures. Scale bars 100 μm. Semi-quantitative expression levels of pS935LRRK2 and pS129αSNCA after 2 (**D**) and 6 (**E**) weeks. *P*-values are calculated using Mann Whitney test, *$p \leq 0.05$. Number of bioreactors: pS935LRRK2 2w, LRRK2-WT 16, LRRK2-G2019S 12; pS129SNCA 2w, LRRK2-WT 31, LRRK2-G2019S 32; pS935LRRK2 6w, LRRK2-WT 42, LRRK2-G2019S 50; pS129SNCA 6w, LRRK2-WT 48, LRRK2-G2019S 34). (**F**) Time-dependent quantification of Zn expressed as ng/ml in flow-through of 3D cultures (Healthy1 and Healthy1-Mut lines) expressed as means ± SEM. The experiment was performed three times and the media of four technical replicates was pulled each time.

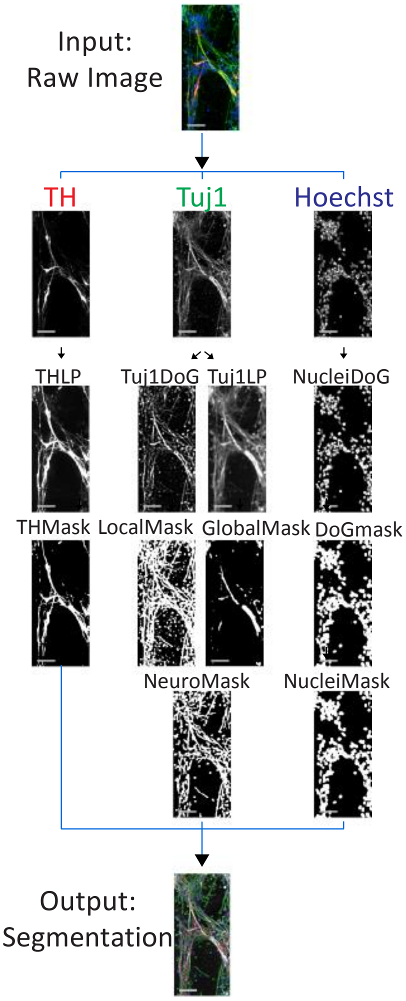

Fig. S2. Key steps of image segmentation. (**A**) For segmentation of neurons, a strategy combining global and local thresholding was implemented. For global thresholding, image preprocessing was done via low pass filtering (Tuj1LP). The raw Tuj1 channel was convolved with a Gaussian filter and the global neuronal mask was defined by threshold 150 (Tuj1GlobalMask). A difference of Gaussians was applied in the preprocessing step (Tuj1DoG). The local neuronal mask was defined by those pixels with values larger than 3 (Tuj1LocalMask). The concepts of global and local thresholding were combined by retaining those pixels in the neuronal mask, which were detected by at least one of these methods (NeuroMask). For the analysis of the TH channel, an additional mask was defined by preprocessing the raw TH channel via convolution (THLP), and thresholding by pixel value 100 (THMask). Image preprocessing for the segmentation of nuclei was done via a difference of Gaussians (NucleiDoG). The first rough nuclei mask was defined by those pixels with graytone values larger than 10 (NucleiDoGmask). Only connected components with at least 200 pixels were retained (NucleiMask).

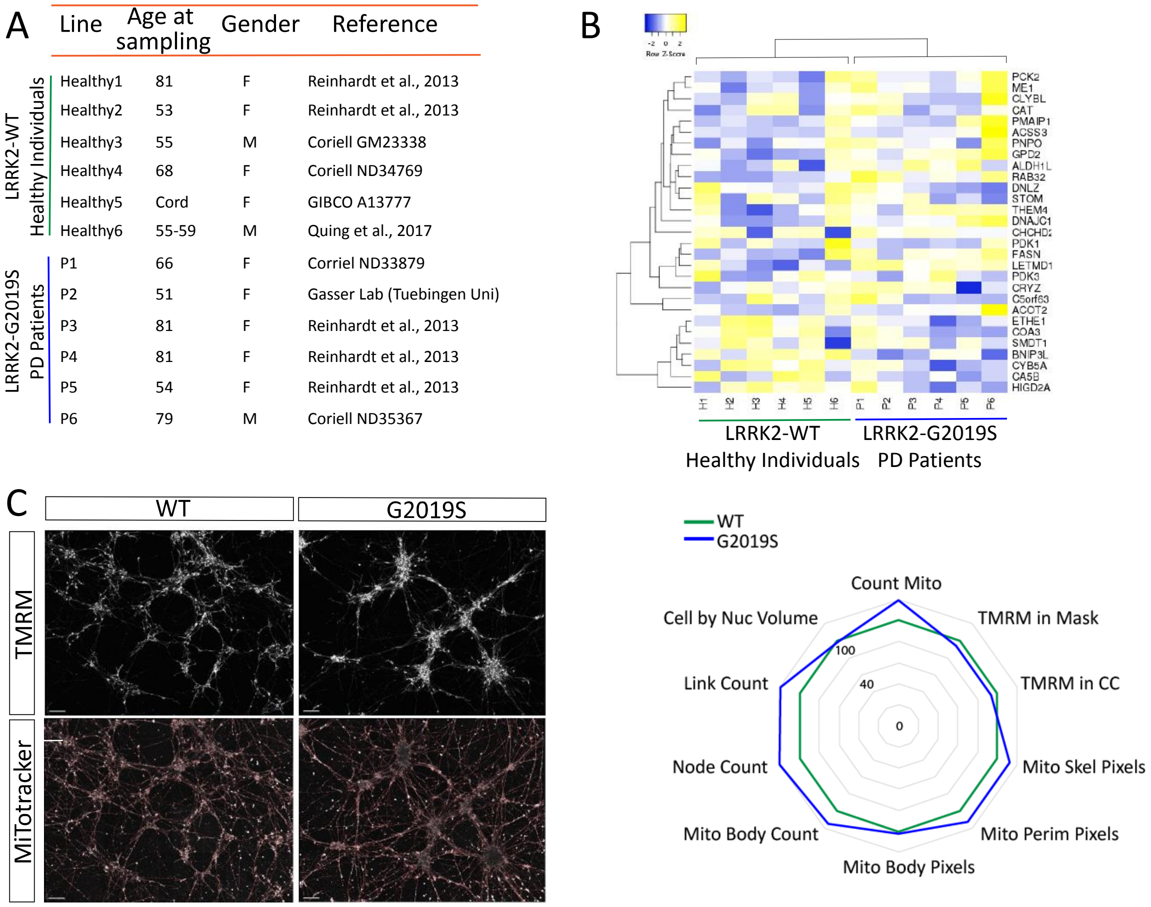

Fig. S3. (**A**) Table illustrating the characteristics of cell lines used for the microarray analysis. (**B**) Heat map visualization of mitochondrial gene expression alterations in PD patients (P1 to P6) in comparison to healthy controls (H1 to H6). Gene expression values have been transformed to Z-scores to facilitate the comparative visualization, and hierarchical clustering was applied to the data in order to group genes with similar alteration patterns (see the dendrogram on the left). (**C**) Representative maximum intensity projection of confocal images of LRRK2-WT and LRRK2-G2019S neurons cultured in 2D for 2w showing TMRM raw channel, and segmented mitochondria (red line) superimposed on Mitotracker green channel. (**D**) Radar plot showing several features extracted from nuclear and mitochondrial segmentation after 2w (number of wells: LRRK2-WT 33, LRRK2-G2019S 33). For the analysis, 45 fields at 20x magnification were acquired for each well (15 fields for 3 planes). No statistical significant difference was observed. The lines used were those described in Fig.S1A.

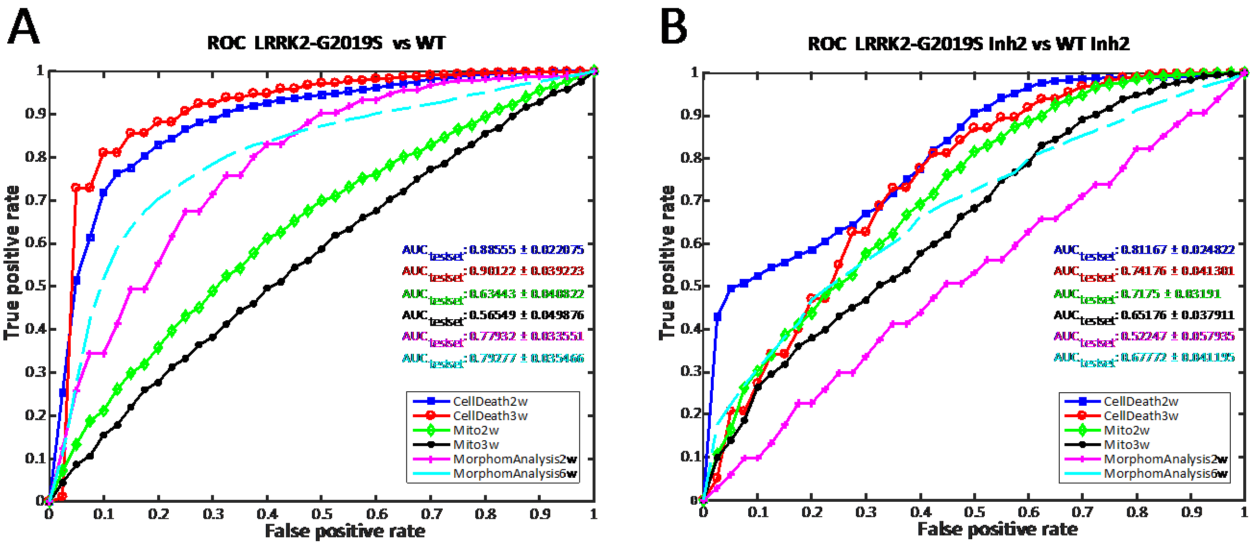

Fig. S4. ROC analysis. (**A**) ROC analysis comparing LRRK2-G2019S versus LRRK2-WT and **(B)** comparing LRRK2-G2019S Inh2 versus LRRK2-WT Inh2 for all different assays at all time-points. The area under the curve (AUC) is indicated.

Table S1: number of samples for the different subgroups in the different assays.

Assay and time point	***# samples Vehicle (G2019S/WT)***	***# samples Inh2 (G2019S/WT)***	***# initial features***
Cell death 2w	**68/66**	**71/64**	11
Cell death 3w	18/22	24/26	11
Mitochondrial assay 2w	**60/68**	**62/54**	21
Mitochondrial assay 3w	**58/63**	**64/53**	21
Morphometric analysis 2w	30/28	29/29	46
Morphometric analysis 6w	32/41	59/45	46
